# Exploring the Validity of the Perceived Restorativeness Soundscape Scale: A Psycholinguistic Approach

**DOI:** 10.3389/fpsyg.2018.02224

**Published:** 2018-11-16

**Authors:** Sarah R. Payne, Catherine Guastavino

**Affiliations:** ^1^The Urban Institute, Heriot-Watt University, Edinburgh, United Kingdom; ^2^School of Information Studies and Centre for Interdisciplinary Research in Music Media and Technology, McGill University, Montreal, QC, Canada

**Keywords:** soundscape, perceived restorativeness scale, perceived restorativeness soundscape scale, attention restoration theory, soundscape assessment, behavior setting, café

## Abstract

Soundscapes affect people's health and well-being and contribute to the perception of environments as restorative. This paper continues the validation process of a previously developed Perceived Restorativeness Soundscape Scale (PRSS). The study takes a novel methodological approach to explore the PRSS face and construct validity by examining the qualitative reasons for participants' numerical responses to the PRSS items. The structure and framing of items are first examined, to produce 44 items which are assessed on a seven-point Likert agreement scale, followed by a free format justification. Ten English speaking participants completed the PRSS interpretation questionnaire in two cafes in Montréal, Canada. Interpretation of participant free format responses led to six themes, which related to either the individual (personal attributes, personal outcomes), the environment (physical environment attributes, soundscape design) or an interaction of the two (behavior setting, normality, and typicality). The themes are discussed in relation to each Attention Restoration Theory (ART) component, namely Fascination, Being-Away, Compatibility, and Extent. The paper concludes by discussing the face and construct validity of the PRSS, as well as the wider methodological and theoretical implications for soundscape and attention restoration research, including the terminology importance in items measuring ART components and the value of all four components in assessing perceived restorativeness.

## Introduction

Soundscapes have the potential to enhance or damage our experience of a place and can have important consequences for people's behavior (e.g., Aletta et al., 2016b; Bild et al., 2016) performance (Clark and Sörqvist, 2012), health and well-being (Stansfeld et al., 2005; World Health Organisation, 2011; Van Kamp et al., 2015). To help design supportive, sustainable environments, soundscape assessment tools are necessary to understand individuals' experiences. An important evaluation criterion for people's experience of some places and its soundscape, is the level of psychological restoration that users may achieve from visiting the place (Gidlöf-Gunnarsson and Öhrström, 2007; Payne, 2008). One form of psychological restoration is attention restoration which refers to individuals' need to recover from attentional fatigue (drained cognitive resources from directed attention) and reflect upon daily or life issues (Kaplan and Kaplan, 1989; Herzog et al., 1997). Restorative environments enable individual users to experience high levels of attention restoration. Assessments of an environment's potential to provide attention restoration can be made using scales assessing the extent an environment is perceived as having the qualities, or components that are theoretically considered important for restoration. Scales such as the Perceived Restorativeness Scale (PRS; Hartig et al., 1997) and the Perceived Restorative Component scale (PRC; Laumann et al., 2001) are commonly used in studies which only present visual cues. To help understand and design soundscapes which enhance restoration, these previous measures of perceived restorativeness were adapted to create a tool to specifically assess the perceived restorativeness of the *soundscape* (Payne, 2013). An important part of creating new tools is to test their reliability and validity. Reliability and partial concurrent validation (getting similar results to existing scales) have previously been demonstrated for the Perceived Restorativeness Soundscape Scale (PRSS) (Payne, 2013). However, public comprehension of scale items was unclear and this affects its face validity (does it measure what it is supposed to?) and construct validity (does it measure the underlying construct?). Therefore, the aim of this paper is to further examine the validity of PRSS items, through a psycholinguistic analysis of participants' free format descriptions which justify their numerical PRSS item ratings.

As reported in Payne (2013) the (PRSS) was developed from the Perceived Restorativeness Scale (PRS; Hartig et al., 1997) and the Perceived Restorative Component scale (PRC) (Laumann et al., 2001). These assessment tools that measure the perceived qualities of an environment in terms of the presence of four theoretical components considered necessary to create a restorative environment and experience. Fascination, Being-Away, Compatibility, and Extent, are the four Attention Restoration Theory (ART) components considered necessary for an environment to be restorative (Kaplan and Kaplan, 1989; Kaplan, 1995). Fascination is a description of involuntary, effortless attention. It is the ability of a stimulus to have attention-holding properties, either without the individual needing to direct attention to focus upon the stimulus, or by inhibiting other stimuli from gaining attention. Being-Away involves a physical or conceptual shift away from the present situation or problems, to a different environment or way of thinking, allowing tired cognitive structures to rest while activating others. Compatibility is the matching of the environment's affordances to the individual's needs and inclinations. The environment needs to be responsive enough to an individual's planned behavior and for the individual to have aims that fit the environment's demands. A high match between the individual and the environment results in the individual using little directed attention as few differences need to be resolved, thus providing opportunities for restoration. An environment with Extent is one that is “rich enough and coherent enough so that it constitutes a whole other world” (Kaplan, 1995, p. 173). Extent has two subcomponents, Coherence and Scope (Kaplan and Kaplan, 1989). Coherence relates to how elements in the environment connect, with their structure and organization combining to make sense (a coherent whole). Scope relates to the scale of the environment (imagined or physical) and quantity of its attributes that the individual is sufficiently engaged.

The PRS and PRC are component-measuring scales which examine the attributes of the person-environment interaction to help determine what makes an environment, or specifically the soundscape in the case of the PRSS, potentially restorative. Understanding the person-environment relationships through these components will enable designers to consider people's perception and behavior in context and elements of the environment that could be enhanced or removed to improve restoration. Taking the individual contextualized approach is in line with the soundscape definition set by the International Organization for Standardization (ISO, 2014, p. 1): “acoustic environments as perceived or experienced, and/or understood by a person or people, in context.”^1^

The PRSS measures the perceived level of these four ART components in relation to an environment's sound through a number of items, each designed to measure one of the four components (Payne, 2013). Developed largely by replacing the word “place” in PRS and PRC items with “sonic environment,”^2^ the PRSS successfully differentiated between soundscapes from different types of environment, the same type of environment, and within the same place (Payne, 2010, 2013). Similar to perceived restorativeness environment scale findings and measures of restorative outcomes for environments (Hartig et al., 1997; Laumann et al., 2001; Herzog et al., 2003; Kahn Jr et al., 2008), the more “natural” the soundscape the more restorative the soundscape was perceived to be Payne (2013). These results in part support concurrent validity of the PRSS as a measure of perceived restorativeness. However, still to be determined, is its face validity which evaluates if it is measuring what we think it is, the restorativeness of *soundscapes* (rather than say visual elements), and its construct validity which evaluates if it's measuring the underlying construct, such as Fascination.

To enhance construct validity, the PRSS and original PRS and PRC items use words relating to the theoretical attention restoration components and their definition. This results in words reflecting the researcher's interpretation and understanding of the relevant concepts, rather than words that the public would normally recognize and use to evaluate a soundscape or place. In turn, the public who are unfamiliar with the concepts being explored, find items in the scale strange and difficult to interpret (Payne, 2013). The wording of items may be particularly problematic for a soundscape scale as people are not used to discussing sounds to the same degree as visual aspects, and in comparison have a limited vocabulary (Dubois, 2000; Guastavino, 2006, 2007; Davies et al., 2013). Therefore, any restorative soundscape measuring tool needs to have simple, comprehensive language that is easy for a respondent to understand.

Examination of the grammatical structure and framing of items within existing perceived restorativeness environment or soundscape scales (Hartig et al., 1997; Laumann et al., 2001; Purcell et al., 2001; Payne, 2013) also highlight a number of inconsistencies. Differences occur between item composition depending on the ART component being assessed, as well as within and between items developed by different authors. Namely, differences exist in (i) the presence or absence of personal pronouns (e.g., I, me), (ii) the location (holistic framing) or elements (specific framing) under discussion (e.g., soundscape vs. sounds), and (iii) the terms used to describe each theoretical ART component through adjectives qualifying the environment (e.g., fascinating) or verbs refering to individual actions (e.g., discover). Each of these are problematic for the face and construct validity of the tool, as differences in item structure and framing of assessment tools can influence respondent's ratings (Bentler et al., 1971; Scott and Canter, 1997). For example, individual items may not be interpreted in the intended manner (low face validity) and items grouped together will therefore not represent their associated ART component but another aspect instead (low construct validity). If public responses are influenced by these psycholinguistic differences, it affects how the PRSS results should be interpreted.

The aim of this paper is to explore public comprehension and interpretation of PRSS items to explore item face and construct validity in assessing the perceived restorativeness of soundscapes. Initially, the paper examines the vocabulary, grammar, and framing of items used within perceived restorativeness scales to develop a “PRSS interpretation questionnaire.” Innovatively, to determine the interpretation of PRSS items this study examines the free-format description used by participants to justify their numerical PRSS ratings, rather than conducting numerical analyses on the provided ratings. Face and construct validity cannot be definitively answered through this psycholinguistic approach as results are, for example, not being tested against comparable previously validated measures of the same concept. However, indications of face validity are expected in terms of participant responses being dominated by reference to sounds, rather than visual features. Whilst construct validity should be indicated from participant responses predominantly referring to terms used to describe their designated ART component, and potentially mention ART outcomes (recover and reflect).

## Methodology

### Psycholinguistic analysis of PRS, PRC, and PRSS items

In a previous study, public respondents previously raised comprehension issues with some PRSS items which had been developed from PRS and PRC items (Payne, 2013). Therefore, this study examined the linguistics of PRS, PRC, and PRSS items. The linguistic examination identified a number of deviations by the PRSS away from the original words used by PRS and PRC items. For example, some of the key theoretical words such as “Fascinating” did not appear in the PRSS, which could reduce the PRSS construct validity. The choice of nouns and adjectives used within items are important as they represent the operationalization of each theoretical ART component and should be influential in respondents' ratings. Therefore, the key nouns and adjectives used should be comprehensible and are vital for construct validity. This issue is not restricted to differences between the perceived restorativeness soundscape scale (PRSS) and environment scales (PRS, PRC). Differences also exist in the descriptive words used in items to assess the same ART component *between* perceived restorativeness environment scales by different authors, as well as differences existing *within* each authors scale of perceived restorativeness. For example, some Fascination items refer to an interpretation of the *content* of the environment, using an adjective (“I find this place *fascinating*”; Hartig et al., 1997) while others explicitly refer to a *process*, using the infinitive verb (“There is much *to explore* and *discover* here,” Hartig et al., 1997). This infers subtle differences in the conceptual processes that the item is measuring and the manner in which the individual interacts with the environment. Both may be important for defining and measuring the concept, or they may be a by-product of the item development through the chosen language (e.g., Swedish, German, French, or English) and word composition, without a full consideration of the implications for the concept measurement. From *what* is being said and *how* it is being said, psycholinguistic analysis can be used to derive inferences about how people process and conceptualize sensory experiences (Dubois, 2000). Examination of the linguistics spontaneously used by participants to justify their numerical responses will determine item comprehension and interpretation in relation to the underlying theoretical component being measured. Specifically, the analysis of the use of personal pronouns can be used to infer different conceptualizations at varying levels of subjectivity. For example, the use of singular first-person pronouns (“I”, “me”) refers to idiosyncratic experiences rather than shared knowledge, the use of collective pronouns (“we”, “us”) refers to negotiated meaning as collective knowledge, and the absence of personal pronouns (e.g., “*it”*) refer to consensual knowledge conceptualized as objective “facts.”

In instructions for completion of the perceived restorativeness environment scales, participants are asked to consider the statements in relation to how much it applies to their experience, through the use of the pronoun “my.” However, when participants are completing questionnaires with numerous items, at times participants may not thoroughly read and take on board all parts of the instructions, thus the emphasis on *their* experience can be missed if it is only referred too in an opening instruction. In other validated scales, where the individual's perspective is required, the items all begin with the words “*I*.” to emphasize the individual experience (e.g., Warwick-Edinburgh Mental Well-being Scale; Tennent et al., 2007). In contrast, there are variations in the use of personal pronouns (“I,” “my”) within and between the sets of PRS and PRC items designed to measure each ART component. Seventy-three percent of the 64 original items examined included a pronoun. Items that include personal pronouns infer that the interaction between the individual and the environment is important for perceiving the restorative qualities of a soundscape. Without the inclusion of a personal pronoun, items could be agreed with *in principle* but does not necessarily mean the individual thinks the soundscape provides restorative qualities for *themselves*. Although this is only a subtle difference, to understand variations in responses from different groups of people, it is important to know exactly how the item is being interpreted and if the personal element is involved in the given rating. Examination of the presence or absence of personal pronouns in individuals' perceived restorativeness responses will indicate the importance of the interaction between the individual and the environment for each ART component.

Finally, as highlighted earlier, the framing of items is influential over participant interpretation and responses (Bentler et al., 1971). PRS, PRC, and PRSS items have been framed in three different ways; holistic, specific, both holistic, and specific. Most items refer to the holistic environment, namely the “soundscape,” “place,” or “setting,” such as “*I find this sonic environment appealing*.” Some items refer to individual or specific elements within an environment, namely the “sounds,” “things,” or “objects,” such as “*When I hear these sounds I feel free from work, routine and responsibilities*.” Occasionally items refer to both the holistic environment and specific elements, such as “*Hearing these sounds hinders what I would want to do in this place*.” Variation in item framing across the different scales may result in their face validity differing. Furthermore, construct validity may suffer if items are framed differently across each component, for example framing Fascination items holistically and Being-Away items specifically, without any theoretical justification for this variation. Further issues arise if items assessing the same component are framed both holistically and specifically, as rating outcomes become harder to interpret. For example, if the result was a low perceived restorativeness rating, to improve the soundscape should an individual sound be removed/altered or is it the combination of sounds that is detrimental? If the framing of the item causes individual respondents to interpret and answer differently, the unsystematic variation in the framing of PRSS items makes it impossible to redesign a soundscape based on the results. Examination of responses to “identical” paired items, either framed holistically or specifically, will identify if both sets of items are easy to interpret, if both are important for evaluating a component, and if interpretation of prior results should be reviewed as item framing has caused variations in responses.

### Development of a PRSS interpretation questionnaire

To examine the public understanding of items evaluating the perceived restorativeness of the soundscape an “interpretation questionnaire” was developed, consisting of items for participants to numerically respond too and provide qualitative justifications for those responses, from which their interpretation of the item can be inferred. Development of the questionnaire was directly based upon a large number of PRS and PRC items as well as PRSS items. This was due to the above observations of differences in PRS, PRC, and PRSS item structure and composition, and the importance of, and consistent use of, PRS and PRC by researchers. In total there were 64 potential perceived restorativeness items from three different scales (*n* = 23, Hartig et al., 1997; *n* = 22, Laumann et al., 2001; *n* = 19, Payne, 2013). To make a feasible questionnaire for participants, the list was reduced to 22 items, which were based upon 35 of the original items (Table 1, column 2). The reduction was achieved by noting similarities in items and removing items that included words referring to a sensory modality (such as “I see”), or ambiguous items using homonyms [words with two meanings; such as “Everything here seems to have a proper place,” with “place” meaning a location within the place/environment, rather than the place (environment) itself]. To avoid affecting construct validity, items using similar words but representing different ART components were also removed (Compatibility: “This sonic environment *fits* with my personal preferences”; Extent: “The sounds I am hearing *fit together* quite naturally with this place”). Additionally, some items were included to ensure a balance between the different types of item compositions used in the original scales for each ART component, such as the use of adjectives or infinitive verbs.

**Table 1:** Relationship between the original PRSS, PRS and PRC items and their adapted versions for the PRSS interpretation questionnaire.

**Keyword**	**Original PRSS, PRS or PRC item**	**Holistic framing**	**Specific framing**
**FASCINATION**			
Curiosity	This place awakens *my* curiosity^a^	This soundscape awakens *my* curiosity	*My* curiosity is awoken by these sounds
Discover	There is much to explore and discover here^a^; There is plenty to discover here^b^	There is plenty for *me* to discover in this soundscape	There are plenty of sounds for *me* to discover
Fascinating	This place is fascinating^a^	*I* find this soundscape fascinating	These sounds, *I* find fascinating
Interest	Following what is going on here really holds *my* interest^a^	Following what is going on in this soundscape really holds *my* interest	*My* interest is really held by following what is going on with these sounds
**BEING-AWAY**			
Break	Spending time here gives *me* a break from *my* day-to-day routine^a^; Listening to these sounds gives *me* a break from *my* day-to-day listening experience^c^	Spending time in this soundscape gives *me* a break from *my* day-to-day routine	*I* get a break from *my* day-to-day routine from spending time with these sounds
Concentration	I experience few demands for concentration when *I* am here^a^	This soundscape demands *my* concentration	*My* concentration is demanded by these sounds
Demands	This is a place to get away from things that usually demand *my* attention^a^ I experience few demands for concentration when *I* am here^a^	*I* experience few attentional demands by this soundscape	From these sounds, *I* experience few attentional demands
Free from	When *I* am here *I* feel free from work and routine^b^; When *I* am here *I* do not need to think of *my* responsibilities^b^; When I hear these sounds *I* feel free from work, routine, and responsibilities^c^	When *I* am in this soundscape *I* feel free from work and/or responsibilities	*I* feel free from work and/or responsibilities when *I* am with these sounds
Obligations	*I* am away from *my* obligations^b^; When *I* am here *I* do not need to think of *my* responsibilities^b^	When *I* am in this soundscape *I* need to think of *my* obligations	*I* need to think of *my* obligations when *I* am with these sounds
Refuge	This place is a refuge from unwanted distractions^a^; This sonic environment is a refuge from unwanted distractions^c^	This soundscape is a refuge for *me* from unwanted distractions	These sounds are a refuge for *me* from unwanted distractions
**COMPATIBILITY**			
Accordance	There is an accordance between what *I* like to do and these surroundings^b^	There is an accordance between what *I* like to do and this soundscape	There is an accordance between these sounds and what *I* like to do
Adapt	*I* rapidly adapt to this setting^b^; *I* rapidly get used to hearing this type of sonic environment^c^	*I* rapidly adapt to this soundscape	*I* rapidly adapt to these sounds
Do what want	It is easy to do what *I* want here^a^	It is easy to do what *I* want while *I* am in this soundscape	While *I* am with these sounds, it is easy to do what *I* want
Fit	Being here fits with *my* personal inclinations^a^; This sonic environment fits with *my* personal preferences^c^	Being in this soundscape fits with *my* personal inclinations	My personal inclinations fits with being with these sounds
**EXTENT—COHERENCE**			
Belong	The existing elements belong here^b^; All the sounds *I'm* hearing belong here^c^	The existing sounds belong to this soundscape	
Fit together	The things and activities *I* see here seem to fit together quite naturally^b^	The sounds fit together to form a coherent soundscape	
Coherent	The surroundings are coherent^b^ All the sounds merge to form a coherent sonic environment^c^	This soundscape is coherent	These sounds are coherent
Organized	It is easy to see how things here are organized^a^	This soundscape is clearly organized	The sounds are clearly organized
Order	There is a clear order in the physical arrangement of this place^a^	There is a clear order in the physical arrangement of this soundscape	The physical arrangement of these sounds has a clear order
**EXTENT—SCOPE**			
Exploration	This place is large enough to allow exploration in many directions^a^	This soundscape is large enough to allow exploration in many directions	There are plenty of sounds to allow exploration in many directions
Limitless	It seems like this place goes on forever^a^; The sonic environment suggests the size of this place is limitless^c^	It seems like the extent of this soundscape is limitless	The extent of these sounds seems limitless
Spacious	*I* experience this place as very spacious^a^	This soundscape feels very spacious	These sounds feel very spacious
Whole world	This place has the quality of being a whole world to itself^a^	This soundscape has the quality of being a whole world to itself	These sounds have the quality of being a whole world to themselves

Personal pronouns are in italics. Author of original item:

a *(Hartig et al., 1997), PRS*;

b *(Laumann et al., 2001), PRC*;

c *(Payne, 2013), PRSS*.

A series of adaptations to the original items (Table 2) were necessary to reflect the psycholinguistic issues identified earlier, namely including personal pronouns when they were missing, and addressing differences in item framing. Therefore, all items, except for Extent items were adapted to include a personal pronoun. Pronouns were not included into Extent items due to its definition which refers to the environment more than the interaction between the environment and an individual. Additionally, there was a major absence of personal pronouns in existing PRS or PRC Extent items; only six of 16 PRS/PRC Extent items include personal pronouns, with none in the Extent items by Laumann et al. (2001). To convert PRS and PRC items to soundscape items, the words “place,” “here,” “setting,” and “surroundings” were changed to “soundscape.” The previously used “sonic environment” in PRSS items was also converted to “soundscape” in line with the new ISO definition (ISO, 2014), and a definition was provided to participants. This initially generated 15 holistically framed items. Specific framed items were then generated by changing the word “soundscape” to “sounds,” before reversing their sentence order to avoid a repetitive feeling for participants, whilst keeping the same item meaning (Table 2). Six items (*refuge, adapt, coherent, clearly organized, spacious, whole world*) however kept the same sentence structure or they became incomprehensive. One Extent item (*belong*) was framed in both a holistic and specific way to avoid a nonsensical item. All items, except two (*obligations* and *concentration*), were positively framed, with high agreement relating to high perceived restorativeness.

**Table 2:** Example of item development for the PRSS interpretation questionnaire.

1.	Original PRS or PRC item	This place is fascinating
2.	Conversion to PRSS item	This soundscape is fascinating
3.	Addition of personal pronoun (holistic framing)	I find this soundscape fascinating
4.	Conversion to PRSS item with specific framing	I find these sounds fascinating
5.	Reverse sentence structure to avoid repetitive feel	These sounds, I find fascinating

The final PRSS interpretation questionnaire consisted of 44 items (Table 1, column Holistic framing and Specific framing). These were 22 paired items which were similar except for their framing being holistic (*soundscape*) or specific (*sounds*), and all included personal pronouns, except for the Extent items.

### Environment

The PRSS interpretation questionnaire and subsequent interviews were conducted in two downtown cafes in Montréal, Canada. An indoor environment was necessary due to weather conditions and cafés are frequented for restoration as well as occasional work, thus providing the potential to show the validity of scale items in an environment that may be restorative to some and not others. This helps test the breadth of the scale comprehension, rather than testing it in a traditional restorative environment, such as a quiet, outdoor green space. To be valuable the PRSS should be comprehensible for studies indoors and outdoors, thus although subtle result differences may arise from using an indoor environment, this study helps extend the range of environments used in restoration studies. Additionally, two cafés were utilized to test the scale across multiple conditions and to avoid results being dependent on the interpretation of items in relation to the specific conditions of one environment. The ability of the PRSS to differentiate within one given context is particularly necessary if it is to be helpful in designing restorative soundscapes, and be of value to restorative environment research which is progressing beyond outdoor natural environments.

**Figure 1:**
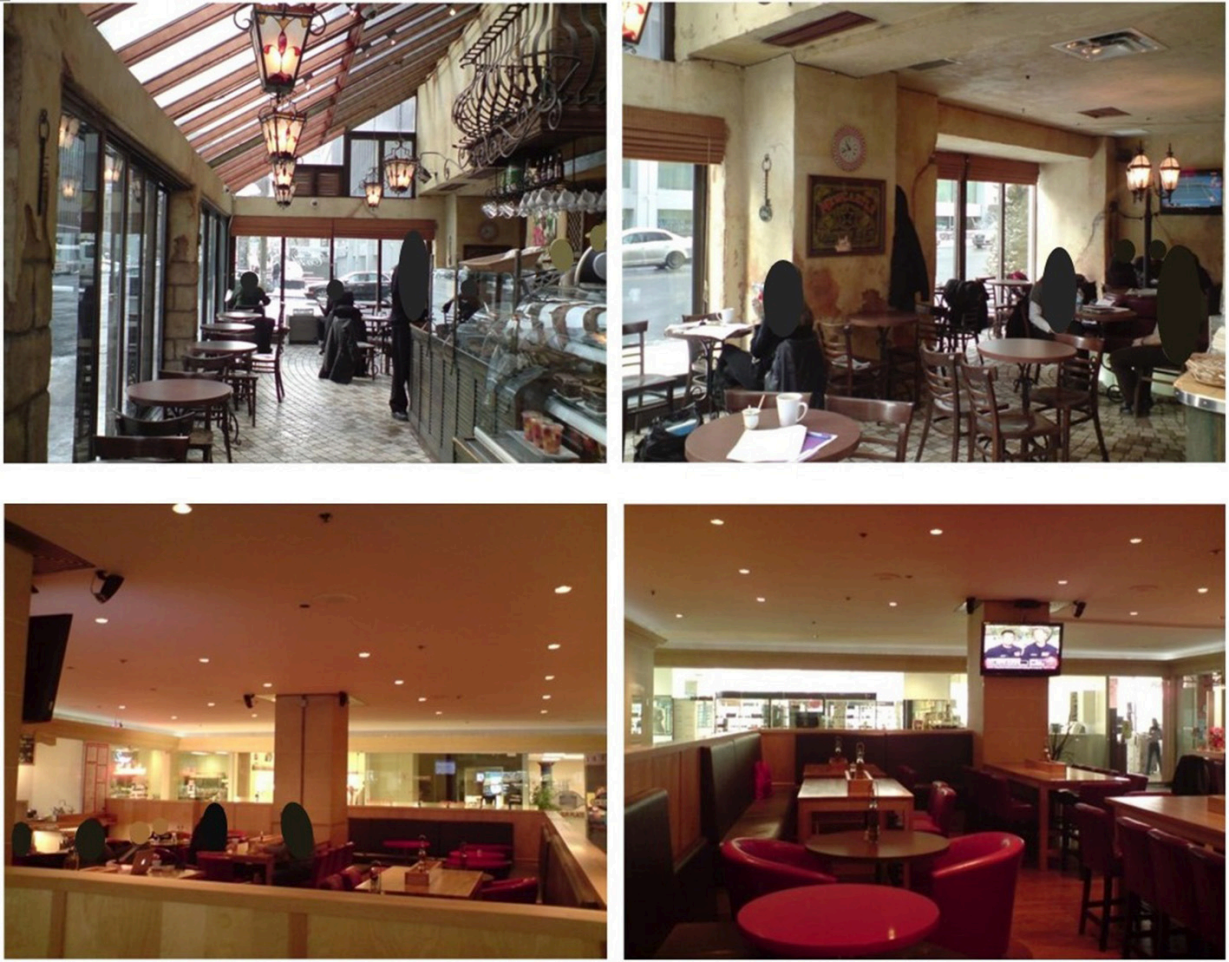
Café A (**Top Two**) and Café B (**Bottom Two**).

The two cafés are located across the road from each other, by offices and a university campus, and were distinctly different (Figure 1). Café A had expansive windows on the outer “wall,” resulting in little need for artificial lighting, and the adjacent busy road and pavement was clearly visible. Overall, it had a rustic theme, basic chairs and tables, as well as a service counter at the entrance displaying food. Café B was enclosed by a small internal wall to separate the café from the surrounding thoroughfare to apartments and a small shopping complex. This café relied on artificial lighting and had considerably fewer customers during interviews than Café A. Overall, it had a modern luxurious theme, large cushioned chairs or stalls at a variety of table types, and an open plan kitchen on one side. Both cafés had a television on with no sound, and pre-recorded music or a radio station played from the array of speakers. Acoustic measurements were not taken as this study is interested in the interpretation of the items, rather than documenting and assessing the perceived restorativeness of the soundscapes in these two cafés.

### Participants

Ten English speaking participants, aged 20–47 years (median = 25–34 years, 70% female) were recruited via public forums. Two participants had slight hearing issues (undiagnosed tinnitus; right ear hears less treble), but both said it did not knowingly affect their results. In general, participants reported being fairly sensitive to noise ($\bar{x}$ = 5.5, s.d. = 1.27, on a 7 point scale) and very aware of sounds ($\bar{x}$ = 5.9, s.d. = 1.45, on a 7 point scale). On average participants visited a café weekly, thus it was a familiar setting. Participants visited cafés for multiple reasons, including for food and drink, or for work, but their main reason was for socializing (*n* = 8).

This study was conducted in accordance with the recommendations of the British Psychological Society and the protocol was approved by the Research Ethics Board II at McGill University. All participants gave written informed consent in accordance with the Declaration of Helsinki.

### Measures

The PRSS interpretation questionnaire consisted of 44 items, which were presented in a random order to each participant. All items were rated on a seven point Likert scale from completely disagree (1) to completely agree (7). Each item was followed by a space to provide the “*reason for your chosen response*.” This paper only explores the reasons for responses rather than the numerical assessment, as the sample size is small and the aim is the interpretation of the items, not the assessment of these café soundscapes.

### Procedure

Participants were recruited via an advertisement for a study on the experience and evaluation of urban places. Questionnaire and interviews were completed on weekdays between 10 a.m. and 12 p.m. (*n* = 3) and 3 and 6 p.m. (*n* = 7). Half of the participants participated in café A, half in café B. Information sheets entitled “The evaluation of soundscapes within urban places and evaluating a soundscape assessment tool” were provided. This included a soundscape definition; “A soundscape is the collection of sounds and subsequent ambience that can be heard within a particular location. It is a holistic aspect, whereby everything together is larger than the sum of its parts. Thus the sum or collection of sounds is more than each individual sound.”

Participants were met within the café, bought a hot drink, invited to review the information sheet again followed by the completion of consent forms. They were then asked to consider the soundscape and sounds for 30 s before listing perceived sounds. The PRSS interpretation questionnaire was then completed. Participants could ask questions at any point and were to underline questions they particularly struggled answering or understanding. A semi-structured interview and demographic questionnaire followed before debriefing. Only free format written responses are analyzed in this paper. Participation lasted around an hour and was recorded on Dictaphones and transcribed. Participants received $10 and a hot drink for taking part.

### Analysis

Participants' written justifications for their numerical ratings were analyzed using the method of constant comparison (Glaser, 1965). Using Nvivo and Excel software Author 1 coded all data (participant responses) and compared notes with Author 2 who separately coded half the data. Coding of individual responses was not mutually exclusive as the 440 responses were coded multiple times on occasions. Both authors produced similar codes with slight variance in the terminology used to name the coding. Discussions and further constant comparison of the data occurred until authors were confident of their interpretation of the data. All components had 80 potential participant responses, except Being-Away which had 120 potential responses, thus percentages, rather than occurrences, are used to compare across components.

## Results and discussion

The results of participants' justifications for their PRSS ratings are presented and discussed below. First, the themes developed from the authors' interpretation of the data is presented. This is followed by a detailed explanation of each theme using data examples, and discusses the implications of the results in relation to the ART components, PRSS validity, and related literature. Participant quotes begin with their numerical response (1–7), followed by their descriptive justification, with the following brackets stating the item keyword (Table 1) and if framed holistically (soundscape) or specifically (sounds).

### Interpretation themes

The qualitative justifications of participants' numerical responses are depicted by six themes (Table 3). Two of the themes relate to the Individual (Personal Attributes, Personal Outcomes), two related to the Environment (Physical Environment Attributes, Soundscape Design), and two are an Interaction of environment and individual perspectives (Behavior Setting, Normality, and Typicality). These themes and their sub-themes are discussed in turn below, followed by a comparison of responses from holistic and specific framed items. Thirteen per cent of item responses were not interpreted as belonging to one of these themes due to; (i) no answer was provided at all (*n* = 7); (ii) no explanation of the numerical value was given (*n* = 23); (iii) the response did not provide an explanation (“not really”; *n* = 23); (iv) or it related to the study task (“Yes I'm doing the survey”; *n* = 6). There were significant differences across all the ART components and the three overarching theme categories of Individual, Environment and Interaction (χ^2^ = 141.8, df = 8, *p* < 0.001). There were more Fascination, Being-Away, and Compatibility item responses themed as Individual than statistically expected and less Environment responses than expected. Compatibility items also had slightly more responses themed as Interaction than expected. In contrast to the other ART components, Extent Scope and Coherence responses were themed more often as relating to the Environment and less about Individual themes than statistically expected.

**Table 3:** Percentage (and frequency) of responses for each ART component per theme.

		**Fascination** (***n*** = **80)**^**a**^		**Being-away** (***n*** = **120)**		**Compatibility** (***n*** = **80)**		**Extent: coherence** (***n*** = **80)**		**Extent: scope** (***n*** = **80)**		**Total** **number of codes**	**Percent of** **all responses (*n* = 440)**
Individual	Personal attributes	24%	(19)	28%	(34)	48%	(38)	8%	(6)	13%	(11)	108	25%
	Personal outcomes	49%	(39)	41%	(49)	63%	(50)	6%	(5)	13%	(10)	153	35%
Environment	Physical environment attributes	26%	(21)	12%	(14)	26%	(21)	31%	(25)	46%	(37)	118	27%
	Soundscape design	3%	(2)	2%	(2)	1%	(1)	40%	(32)	25%	(20)	57	13%
Interaction	Behavior setting	3%	(2)	8%	(10)	30%	(24)	5%	(4)	9%	(7)	47	11%
	Normality/typicality	18%	(14)	3%	(4)	8%	(6)	18%	(14)	5%	(4)	42	10%
												525	121^b^

a *The number of potential participant responses for this component*.

b *Interpretation of item responses into themes were not mutually exclusive, hence total percentage >100*.

### Personal attributes

Personal attributes were referred to in a quarter of participant responses (25%; *n* = 108) with four different subthemes. These related to participants noting their: (i) preferences for certain types of sounds or experiences; (ii) responses may vary depending on their mood, desire, cognitive ability, or activity; (iii) conscious changes in their perception; and (iv) unconscious perceptual changes.

In nearly half of the Compatibility items and a quarter of the Fascination and Being-Away items participants referred to themselves as an important factor in their response rating (Table 3). This emphasizes the importance of individuals' assessment that the soundscape has the restorative qualities of these ART components. They are making judgements about the restorative nature of the soundscape for themselves and not for others, again emphasized by their higher use of personal pronouns in Compatibility and Being-Away responses (see section Framing of Items With Personal Pronouns). Thus, the PRSS can be a measure to compare individual differences in perceived restorativeness across settings as well as collating information from a number of people to monitor trends in soundscapes' perceived restorativeness across different groups of people.

#### Preference

Individual preferences for sounds and activities (*n* = 42) were frequently mentioned from Compatibility items (*n* = 25/42) and sometimes Being-Away items (*n* = 12/42). Phrasing of the Compatibility items *accordance* and *fit* encourage participants to reflect upon what soundscapes and activities they like in general. Participant responses showed successful contemplation around whether the café soundscape matched those preferences [“6 I prefer this sort of soundscape to one that is too quiet or too loud, like a library or a club/bar” (*fits soundscape*)] or did not match [“2 No, I generally prefer quiet time away from people, unless it's people I've chosen to be with.” (*accordance soundscape*)]. Previous studies have found preferred environments tend to also be perceived as restorative environments, with particularly high Compatibility PRS ratings for favorite places (Korpela and Hartig, 1996). This study's results also suggest high compatibility scores in the PRSS may also relate to favorite soundscapes. Examination of the phrasing and participant responses suggest the relationship between preferred and restorative environments may partly be an artifact of the measuring tool, as half of the Compatibility items are indeed measuring preference, thus associations with restorativeness are to be expected. Indeed part of the Compatibility definition relates to preference as it refers to “one's inclinations” and “fitting to what one would like to do” (Kaplan, 1995, p. 173). Given preference is a common assessment in soundscape studies, further consideration of the relationship between preferred and restorative soundscapes could be explored.

Only one response to the accordance item did not refer to sounds, suggesting the comprehension and face validity was good. In contrast, 6 out of 10 responses to the *fits* item did not directly refer to sounds or soundscapes suggesting it was not assessing the compatibility of the soundscape. Instead, other features of the environment were referred too, such as “5 I like cafes” (*fits sounds*) and “1 If I were to spend time and money in a cafe environment, I would choose one with more character and windows” (*fits soundscape*). Given the wording within the fits item of “personal inclinations” has previously been noted as confusing by participants (Payne, 2013), adaptations to improve or remove this item seem necessary.

#### Depends on mood, desire, cognitive ability, or activity

In this study, participants were sitting in the café and considering the restorativeness of the soundscape due to the task, rather than purposefully having chosen to come to this environment to restore. The consequence of this artificial arrangement meant it made it trickier for participants to provide a fair rating, resulting in many middle numerical ratings of four; “4 Potentially—possibly. But normally it'd be a soundscape I turn off a bit from, in order to concentrate on something else.” (*discover soundscape*). People need restoration for different reasons at different times, and different soundscapes may support these needs or hinder it and may vary depending on the specifics of the scenario that caused the need for restoration. Therefore perhaps unsurprisingly this resulted in participants frequently responding with the statement “it depends” (*n* = 49) particularly for Being-Away (*n* = 23) and Compatibility items (*n* = 14). For example “4 Depends on my level of distractability” (*Attentional demands soundscape*) and “5 Well, I've got things on my mind at the moment, so yes. But at other times, I'd disagree more with this statement” (*Obligations sounds*). Knowing how a person is feeling, their level and type of fatigue (if any) prior to doing ratings would help understand the reasoning behind responses and how this varies the perceived restorativeness qualities.

The environment the study was conducted in, cafés, are also multi-purpose environments with the potential to both work or relax, which may have exaggerated the “it depends” issue; “4 In this soundscape, if I were here to relax, I would feel relaxed. If I were here to work I would probably feel as stressed as my mindset was.” (*free from soundscape*). Unlike other studies where participants are either purposively fatigued beforehand, such as partaking in a lecture (Laumann et al., 2001) or asked to imagine a scenario where they are fatigued and need restoration (Staats et al., 2003), this study did not provide any such situation. Perhaps if a scenario had been provided the “it depends” variation would have been reduced. However, these responses highlight that both Being-Away and Compatibility items are encouraging the individual to consider the restorative qualities of a soundscape at a particular point in time and it may be useful to understand that context fully to understand the reported level of the perceived restorative qualities of the soundscape. This is highlighted by the response to a Being-Away item where the relative differences between the previously experienced and current soundscape being assessed is important in understanding the response: “3 It certainly is if I'm coming off the street; not so much if I'm coming from my home” (refuge soundscape). These relative differences are particularly important to consider when making planning decisions as the relative difference between previously exposed soundscapes and the soundscape under investigation may be important for defining the soundscape as restorative.

#### Conscious perceptual changes

Responses from eight of the Fascination items raise interesting points about how environmental assessments can change over a period of time; “2 After acclimatizing to this environment the sounds begin to feel uniform” (*discover soundscape*); “5 only initially → once they are identified, I'd rather they disappear” (*curiosity sounds*). In this study participants were in the soundscape for around 10 min before they started assessing the soundscape which took around 30 min. Thus, they had prolonged “exposure” to the soundscape which meant their initial assessments could change over time. This allows for the addition of new sounds which may increase fascination, but in this instance, the fascination actually waned over time. In contrast, most laboratory soundscape studies present stimuli for 15, 20, or 30 s (e.g., Carles et al., 1999; Dubois, 2000; Guastavino, 2007; Axelsson et al., 2010) with only a few including longer recordings of 5 min (e.g., Guastavino et al., 2005) before requesting an evaluation of the sound. The results from this study question the suitability of brief exposures to stimuli to make restorative assessment judgements and perhaps other environmental assessment criteria.

#### Unconscious perceptual changes

On an equal number of occasions to the conscious perceptual changes (*n* = 14), participants referred to unconsciously directed perceptual changes (*n* = 14), where “2 no, elements seem to ebb in and out of importance within the soundscape” (*clear order soundscape*). These unconscious variations appeared more in the response to Extent items (*n* = 7/14) suggesting stimuli variations help define the extent of a soundscape; “5 Yes, but now that I'm hearing the outside a little more, that world has expanded to include the street” (*whole world sounds*).

### Personal outcomes

The likely personal outcomes from experiencing the soundscape was the most dominant participant response (*n* = 153). These included subthemes of attention, other cognitive aspects, behavioral actions, and emotions. The PRSS was designed to measure the perceived restorative qualities of the soundscape and thus the likelihood of an individual being psychologically restored, particularly in relation to directed attention, after experiencing a given soundscape. Two specific types of restorative outcomes identified are the ability to recover and reflect (Kaplan and Kaplan, 1989; Herzog et al., 1997). Thus, responses should and did display participants' consideration of what may happen from experiencing the soundscape, with particular reference to attention, recovering, and reflecting. Indeed, over half of the responses to Compatibility items (63%) and nearly half of the Fascination (49%) and Being-Away (41%) item responses mentioned “personal outcomes” from experiencing the soundscape (Table 3). In contrast, Extent items hardly referred to outcomes. Personal Outcomes was the most coded theme, which supports the overall aim of the scale measuring what is likely to happen from experiencing the soundscape, and in part supports its content validity (fair representation of the topic).

#### Attention

Over half of the comments regarding outcomes from the experience were attention related (*n* = 81/153), thus they partially support the role of the PRSS in measuring the attentional qualities of the soundscape (face validity). The PRSS is specifically designed to measure the degree to which directed and involuntary attention is likely to be activated, particularly through the ART component Fascination, which is defined as involuntary effortless attention (Kaplan and Kaplan, 1989; Kaplan, 1995). The majority of Fascination items coded as personal outcomes referred to attention in some form (*n* = 29/39), with some responses directly using the word attention; “2; I don't find it particularly attention grabbing at all” (*fascinating soundscape*). The ability to ignore or tune in and out of attending to the sounds was occasionally referred to, such as; “3; I'm generally curious of the origin of sounds/how they shift etc. But there is a uniformity that is also easy to tune out” (*interest sounds*). Participants' “search” for stimuli that evoked involuntary attention was also associated with a level of interest in the sounds and soundscapes; “3; Fascinating in their transparency and interaction, but I am bored of it and look forward to other soundscapes” (*fascinating sounds*); “1; none of the sounds hold my interest, just my attention most of the time” (*interest soundscape*). Therefore, although the cafe sound/scape did not necessarily invoke positive involuntary attention for some participants, their responses suggest these PRSS Fascination items have construct validity in the sense that they assess the extent of specific types of attention being activated. However, their responses also highlight that involuntary attention may be produced by unwanted and undesirable stimuli which would not be restorative. This is in line with early critiques that negatively evaluated nature, like snakes, can induce involuntary attention, and thus “Fascination” alone cannot result in restoration (Ulrich et al., 1991). Thus, positive associated words are important to include in items measuring Fascination, such as “interest.” Additionally, in line with the positively framed word “Fascination,” originally chosen by the Kaplans to represent involuntary attention (Kaplan and Kaplan, 1989), its definition should always include a positive word, such as “desirable.” This explicit emphasis would assist in the development of valid items for measuring Fascination; for example “There is plenty for me to discover in this soundscape” should become “There is plenty *I want* to discover in this soundscape.” Indeed, researchers have previously noted that when measuring Fascination, three dimensions should be emphasized, namely pleasantness, intensity (amount of effort), and functionality (recover and/or reflect), and in part have been proposed to differentiate between Hard and Soft Fascination (Hartig et al., 1997; Herzog et al., 1997).

For responses relating to outcomes from the experience, Attention was the second most coded personal outcome for Being-Away items (*n* = 29/49) and for Compatibility items (*n* = 18/50). Half of the personal outcome codes for Extent-Scope were for attention but there were very few of them (*n* = 4). Given there is a Being-Away item on “*attentional demands*” it is hardly surprising attentional aspects were often referred too. However, as with Fascination items, participant comments suggest involuntary attention being invoked, but not in a positive way for this soundscape; “1, The sounds themselves are unwanted distractions!” and “1, These are unwanted distractions and I crave the refuge of my own company, space and voice” (*refuge sounds)*. Involuntary attention is generally discussed in terms of positive attributes in relation to restoration, however, in both the Fascination and Being-Away responses, participants make it clear that their attention at times is being demanded by the sounds, whether they like it or not. This recalls that although Fascination may often be defined as “involuntary, effortless attention,” involuntary attention is not equal to Fascination. Unlike visual perception, where an individual can generally choose the direction of their gaze and what they want to look at, audio perception is harder to control and sounds have to be continually filtered and processed to ignore some auditory streams and focus on others (Moore, 2003). This implies another important word in the definition of Fascination, alongside desirable, is “effortless,” which was a key aspect of work by James from which the ART evolved (Kaplan and Kaplan, 1989; Kaplan, 1995). Reemphasising effortless attention, and perhaps the distinction between Hard and Soft Fascination (Herzog et al., 1997), may also place a greater importance on the component Extent—Coherence as a coherent environment would aid effortless attention. Indeed a close relationship between Fascination and Extent has previously been hypothesized, albeit in the opposite direction; a fascinating environment would contribute to a sense of extent (Hartig et al., 1997). Additionally, Extent may therefore be important in differentiating between environments or soundscapes with Hard and Soft Fascination if they vary in degrees of intensity (effort) needed. This is important as Coherence, along with Scope, is sometimes not considered in some restorative environment studies (e.g., Nordh et al., 2009; Lindal and Hartig, 2013) but yet may still be an important component for restoration—particularly restorative soundscapes.

As with many psychological processes, it is hard to study the natural conditions of what is occurring and the influence on the individual, as focussing on the topic causes the individual to think or behave differently. The very nature of the PRSS requests participants to focus on and consider the soundscape thereby activating directed attention; sounds that participants would otherwise have been able to “tune out,” may now take prominence and “demand attention.” Additionally, the process of active listening, which is closer to *musical listening* than the more usual *everyday listening* (Gaver, 1993a,b), clearly influenced some participants' responses. For example, one participant provided a high Fascination rating due to the “interesting” study *process* of active listening and engaging *directed* attention rather than *involuntary* attention, and not because of interesting sounds; “6, Yes, they are ordinary, but it is interesting to be attentive” (*fascinating sounds*). Therefore, although the discussion of attention by participants helps validate PRSS items, it should be remembered that participants' attentional responses during PRSS completion may be different to usual.

#### Other cognitive aspects

Related to attention, participants also noted their ability to concentrate, be focussed, and productive, when in this type of soundscape; “7, Absolutely. I need sounds to keep me focussed” (*refuge sounds*). For others, they referred to the degree the soundscape allowed their thoughts to wander [“2, Not especially, I think my thoughts could drift off” (*concentration sounds*)] or even a wandering exploration of the sounds [“6, If it is busy it allows for my ears to wander; however if it is slow then I tend to keep to my own thoughts” (*exploration sounds*)]. This connects to one of the main (but often neglected in research) outcomes from a restorative environment—reflection (Kaplan and Kaplan, 1989; Herzog et al., 1997). Two Being-Away items, *obligations* and *free from* particularly led to statements about “thinking” and “reflecting”; “5, They did torrent through my head as I waited (obligations soundscape)”; “2, Reminiscent of studying at cafes during undergrad” (free from sounds). However, some questioned whether it was the sounds/cape that was causing this or if the holistic environment caused it instead “4, Sitting in a cafe usually causes me to reflect on my obligations but I would not say the sounds make me” (obligations sounds). This raises face validity issues as it highlights some concerns over the ability for people to answer questions specifically about the soundscape without influence from other sensory stimuli. The soundscape definition includes “in context” (ISO, 2014), thus other sensory stimuli should be included in soundscape assessments. This is in line with current multisensory research showing the interaction between sensory modalities, with sensory stimuli presented in one modality impacting our sensory experiences of another modality (Bayne and Spence, 2015) including the impact of sound on visual landscape assessments (Carles et al., 1999).

Overall, Being-Away items particularly mentioned these other cognitive aspects (*n* = 34/49). This meant that along with the comments relating to Attention, half of all the participant responses to Being-Away items were about cognitive outcomes (*n* = 61/120). The words used in the items such as “think” (*obligations*) also help direct participants to consider reflective aspects. These participant responses support the face validity of the PRSS items in evaluating the soundscapes' potential for providing attention restoration, however some responses question if the focus on soundscapes is appropriate for assessing the involvement of Being-Away in providing attention restoration outcomes.

#### Action

The behavior or activity participants would do because of the soundscape was mentioned in 41 responses (27% of personal outcome responses). Largely, these included participants' ability to do their desired activity, such as reading, talking, and working. Importantly, participants mentioned the action of relaxing which is often associated with recovery (six from Compatibility items, three from Being-Away items), sometimes as the result of other behavioral actions; “5, Socializing and work are both pleasant and relaxing to do here. If occasionally it's distracting” (*accordance soundscape*). Participants had split views on their ability to relax in this soundscape and the task and particular environment may have prevented the word “relax” from being mentioned more frequently by participants. Reference to relaxation and discussing whether it was possible or not, helps validate that the PRSS items were activating restorativeness assessments. This supports the PRSS validity as an instrument to assess the soundscape qualities as those that could produce restorative outcomes of relaxing and recovery (see cognition above).

Overall, when Compatibility items produced responses relating to personal outcomes, they were more frequently about Actions (*n* = 25/50), and Compatibility items also had more action responses than any other ART component. Given the definition of Compatibility, being a match between the environment's affordances and the individuals' needs and planned behavior (Kaplan and Kaplan, 1989; Kaplan, 1995), it is positive that so many desired actions are mentioned in the responses to justify their ratings. This is in addition to the consequences of those actions, such as relaxing, and the impact on their emotions and attention, which were also frequently mentioned in Compatibility items. The diverse spread of responses across three of the four personal outcome subthemes (attention, emotive, and action) suggest that Compatibility items are an important component for the scale and for ART. It draws on all aspects of the theory, with the activation of attention depending on the environment's match to the individual's need and the potential for restorative outcomes.

#### Emotive

Participants at times described the valence of the experience, with Compatibility items responsible for nearly half of the emotive comments (*n* = 16/34). The pleasantness, annoyance, and comfort of the sounds were particularly mentioned; “5 Yes, but the more I listen, the more I'm becoming irritated” (*adapt soundscape*); “7, I am very comfortable in these sounds (*fit soundscape*).” These responses suggest that the soundscape matching the participants' desired emotional mood is important for the individual. Thus emotional aspects are being partly assessed with the PRSS, and support the identified relationship between preferred environments and restorative environments (Korpela and Hartig, 1996) as also discussed earlier. Together the results suggest restorative environments, as assessed by the PRS, PRC, and PRSS, are influenced by emotional responses, however, emotions still play a small role compared to attention and other cognitive outcomes.

### Physical environment attributes

A quarter of participant responses included references to specific physical environment attributes within the environment, such as describing sounds by their sources (*n* = 91), the size of the environment (*n* = 12), or visual elements (*n* = 4). There were also references to present sounds changing over time (*n* = 20).

As there are numerous individual sounds that can be listed, this response theme was the second most frequent to occur, as participants only needed to refer to one sound or visual attribute to be coded here. As found with previous linguistic observations of soundscape work, people tended to describe sound sources rather than sounds (Dubois, 2000). Sounds listed by participants included building services (e.g., ventilation), café related objects (e.g., coffee machine), entertainment systems (e.g., music), people (e.g., talking), external street sounds (e.g., traffic) and a few referring to an “ambience.” Considered sounds will of course vary depending on the environment and the soundscape being assessed, but the presence of this theme highlights that people did consider the individual sound sources that comprise the soundscape. The reference to sounds changing over time emphasized the Extent of the soundscape to participants [n = 11 Extent item responses; “6 It is continually shifting in terms of the composition and nature of the sounds” (*limitless soundscape*)], and its long-term ability for Fascination to remain [*n* = 6 Fascination item responses; “5 lots of new sounds being introduced” (*discover soundscape*)]. Together, the listing of sounds in the environment and their variation overtime, help validate the PRSS, as the variation of sounds between the two café environments and within them at different times was affecting the perceived restorativeness rating of the soundscape. The prolonged exposure to the stimuli (real world soundscape) in this study also provided the opportunity to note the variations in sound sources, and this prolonged exposure has helped the rating of Extent items and some Fascination items. This again suggests the importance for longer stimuli exposure times in laboratory studies to ensure realistic ratings are provided. Extent items are excluded in a number of online and laboratory studies (e.g., Nordh et al., 2009; Lindal and Hartig, 2013) as prior studies have found Extent results do not compare well with the other components, thus questioning the importance of Extent in restoration, while others have critiqued the items for being unrepresentative of the definition (Pals et al., 2009). However, participant responses in this study suggest Extent items are good measures of the perceived restorativeness of the physical environment attributes. It may just be that participants need longer exposure periods to provide valid ratings for Extent, and this has not been noted due to the tendency to use shorter stimuli exposure in lab settings.

Visual attributes such as “blank walls,” “café menu,” and “no windows” were infrequently noted by participants as well as the size of the environment; “1 it feels neither spacious nor crowded I like the enclosed café area which feels private while I'm inside it and prevents people accidently (or not) wandering through on the way elsewhere” (*spacious soundscape*). Although it only occurred on a few occasions, references to non-sound related elements highlights difficulties in translating some perceived restorativeness environment scale items to become sound specific (for the PRSS) rather than a focus on all elements of the environment, as with the PRS and PRC. Therefore, there is still further work to increase face validity of some PRSS and to ensure the items are framed in a way that enables the assessor to focus only on the soundscape if the intention is to assess the perceived restorativeness of the soundscape.

Physical Environment Attributes were particularly mentioned by items assessing Extent (31% by Extent-Coherence and 46% by Extent-Scope items). Thus, what the soundscape is perceived to comprise, and the size of the environment has a strong influence over Extent ratings. The composition of the soundscape (discussed in relation to which sounds are present) and the size of the environment clearly relate to the definition of Extent Coherence (structure and organization) and Extent Scope (scale of environment) (Kaplan and Kaplan, 1989). This suggests that the PRSS items are a good measure of the concept, albeit sometimes currently with an influence from non-sound related elements.

### Soundscape design

Responses from Extent items also predominated the noted theme of Soundscape Design, where the design of the soundscape may have been intentional or not (91% of these responses were Extent items). These largely related to instances of the location of sounds [“3 not really, most sounds seem to emanate from one localized area” (*exploration sounds*)], distances between sound sources [“5 the room ‘feels’ big, sounds are coming from different distances from one another…” (*exploration soundscape*)] or the composition of the sounds and the environment, [“7 absolutely, the decor and the music go well together and the sounds of people passing are hardly noticeable” (*fit together sound to soundscape*); “2 not organized, at all -> they occur independently of any plan” (*organized sounds*)]. References to sounds being in the foreground and background were also made (*n* = 9); “6 foreground/background clearly defined” (*order sounds*). Although this can depend on the individual perceiver rather than the soundscape design, participants referred to it as if it was an objective description of the acoustic environment rather than having the potential to vary by perceptual differences “1 no the sounds clash although the radio is dominant” (*coherent soundscape*).

Soundscape design is an important area of growing interest (Andringa et al., 2013; Kang et al., 2016) to help reduce the negative effects of environmental noise (World Health Organisation, 2011) and potentially consider the positive impacts soundscapes can also have (Davies et al., 2009). Therefore, it is valuable to note the design of the soundscape is considered within the Extent item responses. The Extent items seem to particularly assess the perceived restorativeness of the physical environment attributes with little influence in the potential variation that may arise between individuals (e.g., little reference to personal attributes and outcomes from Extent items, and little use of personal pronouns). In a future study, it would be interesting to examine statistical responses to Extent items from a large number of people's assessment of the same soundscape. If there was little variation, then these items could be used by independent evaluators to help with assessing and designing restorative soundscapes, without the need for large-scale surveys. The words used within the Extent items also has similarities to words frequently used to assess soundscapes in other studies. For example, ART research uses the words “coherent,” “order,” and “spacious,” while soundscape assessment research uses the words “congruence,” “organized,” “harmonious,” “nearby/far,” and “open” (e.g., Carles et al., 1999; Raimbault et al., 2003; Ge and Hokao, 2005; Axelsson et al., 2010).

### Behavior setting

Behavior settings is the interplay between behavior episodes (goal-directed actions), social inputs, and environmental force units (combination of distinct environmental inputs) (Barker, 1965); behavior settings are the physical environment where standing patterns of behavior occur independent of individuals' perception (Schoggen, 1989). In short, a setting where a series of known activities and behaviors would be conducted. The theme of behavior settings emerged in participants' responses as they often referred to “this type of place” and “in an environment such as this,” or quite simply “6 yup café” (*fit together sound to soundscape*) (*n* = 30). Similar to personal outcomes, activities that occurred in the café were mentioned but responses were coded here when they particularly referred to the activity being in this setting, such as “4 I often associate these environments with work/studying and/or planning things in my life - however I do associate it with socializing as well” (*free from soundscapes*). There tended to be a focus on the environment overall rather than a particular consideration of the sounds or soundscapes which only occurred a few time (*n* = 8/30); “3 I feel this type of sound is associated for me with the type of space - cafe - which I do not usually think of as spacious” (*spacious sounds*). It is the matching of both the activity and the physical environment that explains why half of the responses coded in this theme are from Compatibility items, in line with its definition. Therefore the wording of the PRSS Compatibility items successfully induce people to consider both the environment and intended activities, however the intention of the PRSS is supposed to be on the soundscape's affordances, rather than the general environment, questioning its face validity. The soundscape definition refers to the context of the perceiver (ISO, 2014), thus research is increasingly focussing on the activity of the soundscape assessor (Aletta et al., 2016b; Kang et al., 2016). Thus, although Compatibility item responses do question if the soundscape was focussed on, the inclusion of activity focussed items (via Compatibility) will still be important for assessing perceived restorativeness of soundscapes.

On a number of occasions participants ratings were based upon comparisons of this type of soundscape, a café soundscape, with other cafés' or other environments' soundscapes (*n* = 9). For example, “1 I prefer a quieter place to read without distraction and don't drink coffee too often, mainly when the weather is cold” (*accordance soundscape*) and “6 I prefer this sort of soundscape to one that is too quiet or too loud, like a library or a club/bar” (*fit soundscape*). On other occasions participants contrasted the café *environment* to other cafes or environments with no reference to the sound (*n* = 6); “1 If I were to spend time and money in a cafe environment, I would choose one with more character and windows” (*fit soundscape*). These comparisons highlight the choices people usually make regarding where they go to do certain activities, feel certain things, and to have certain outcomes resulting in choosing one behavior setting over another or choices within a type of behavior setting. This supports prior findings that PRSS is sensitive enough to differentiate soundscapes between environments, such as rural, urban park, and city center, and within the same environment type (Payne, 2013). However, questions remain as to whether participants can truly consider the restorativeness of the soundscape without all other aspects of the behavior setting influencing their ratings. As behavior settings of the same type, say café, will produce similar sounds as there will be similar activities and objects in each place, this is understandable, but the interplay of these aspects should be acknowledged when reporting PRSS results. Therefore, it may only be valuable to compare PRSS ratings from the same behavior setting rather than across behavior settings, to avoid non-soundscape aspects strongly influencing the comparative results.

### Normality, typicality, expected, and familiarity

Ten per cent of coded responses referred to the normality of the sounds or soundscapes, and the typicality of them for a café, thus they were sounds they expected to hear there, and that it was a familiar soundscape or environment (*n* = 42). A third of these responses (*n* = 14) came from Fascination items and another third from Extent-Coherence items (Table 3). Two sub themes emerged, with the normality, typicality, expectedness or familiarity referring to the behavior setting (n = 29/42) or referring to individual physical environment attributes (*n* = 13/42). All of the behavior setting subtheme responses were in addition to the main Behavior Setting theme (i.e., mutually exclusive), as were eight of the physical environment attributes sub theme responses in addition to the main Physical Environment Attributes theme. Despite the alignment with other themes, conceptually this theme was interesting to discuss and remain separately. A previous study has also identified familiarity as one of three basic dimensions in soundscape perception (along with Pleasantness and Eventfulness) (Axelsson et al., 2010).

The expectedness of sounds for this study's behavior setting, a café, justified a number of Extent-Coherence items' ratings (*n* = 14/42); “6 sounds I would expect to hear at a café” (*coherent sounds*); “7 Very typical sounds for a cafe. Music, coffee, conversations” (*belong sounds to soundscape*). Thereby the interpretation of coherence was partly about the relationship between the sound and the behavior setting. This may also overlap with a consideration of the Soundscape Design theme which was also predominantly interpreted from Extent-Coherence items.

Fascination was defined earlier as the ability of a stimulus to have attention-holding properties, either without the individual needing to direct attention to focus upon the stimulus, or by inhibiting other stimuli from gaining attention (Kaplan and Kaplan, 1989; Kaplan, 1995). The normality, typicality, expectedness, familiarity of the sounds and behavior setting contributed to participants rating of the stimulus holding their attention. In this instance, it generally resulted in negative ratings [“2 Nothing out of the ordinary happening” (*interest sounds*); “2 I find expected, typical” (*fascinating sounds*)] apart from the positive novel listening experience caused by the study task itself. These negative ratings again suggest the interpretation of Fascination items as relating to *desirable* effortless attention holding stimuli. This is understandable given the wording of most of the Fascination items (*fascinating, curious, interest*) but should be emphasized in the main definition. Familiarity may also be an important aspect to include again in future soundscape studies (see Steffens et al., 2017 for further investigation of the effect of familiarity on soundscape assessments).

### Framing of items as holistic and specific

Comparison of item responses to items framed in relation to the sounds (specific), or related to the soundscape (holistic; Table 4) found no significant differences in the frequency with which they were interpreted as part of a theme (χ^2^ = 1.81, df = 5, *p* = 0.88). This is in agreement with comparisons of the numerical ratings of each set of matched specific and holistic items, that showed little variation (median difference of 1, with 41% of identical responses) (Payne and Guastavino, 2013). This suggests that the framing of the question in holistic or specific terms did not have a strong influence on participants' interpretations.

**Table 4:** Frequency of responses for sounds or soundscape framing items per theme.

		**Framing**	
		**Sounds**	**Soundscape**
Theme	Personal attributes	65	56
	Personal outcomes	87	88
	Physical environment attributes	65	56
	Soundscape design	24	30
	Behavior setting	23	26
	Normality/typicality	17	16

### Framing of items with personal pronouns

There was little variation between participant's individual responses to the use of personal pronouns in holistic or specific framed question, with some having none, and two participants mentioning personal pronouns four times more in holistic than specific framed questions. There was a much larger variation across participants though, with one individual only using personal pronouns four out of the 44 potential responses, whilst the other participants used them between 15 and 30 times. Of greater importance was the variance in the use of personal pronouns across ART component item responses. Personal pronouns were used in two thirds of the responses for Compatibility items (66%), and over half of the time for Being-Away items (58%). This is in line with the high level of Compatibility and Being-Away item responses interpreted as relating to Personal Attributes, with the *individual* being an important aspect of the assessment process. Personal pronouns were used 41% of the time for Fascination items, which is surprising given a high number of responses relating to personal outcomes. Instead participants tended to say “it's interesting” or “it's boring,” perhaps assuming that other people, like themselves, would also rate things similarly (consensual knowledge). Extent Coherence and Extent Scope item responses only included personal pronouns 28 and 38% of the time, respectively. This emphasizes again participants interpretation of the Extent items as less about the individual's assessment of the soundscape, and more of a consensual knowledge conceptualized as an “objective” assessment of the physical environment attributes. Given personal pronouns were excluded from the PRSS Extent items, this may have influenced the results, however, as some participants did respond using personal pronouns occasionally, this suggests the lack of personal pronouns did not completely direct how an individual should respond to the question.

### Sound or vision leading responses

Face validity of the items is generally supported, in this respect, as only on two occasions did participants specifically use visual terms in their responses (“see”), compared to the multiple times participants used acoustic terms (“hear,” “listen,” or “eavesdrop”; *n* = 12, 13, 2, respectively). Participants also considered the predominance of the sounds as either “foreground/background” or “tuning in” on particular sounds (*n* = 10, 8, respectively). This suggests the focus on acoustics rather than visual features was consciously adhered too, however other sensory aspects may have unconsciously affected participants ratings, particularly when behavior setting aspects predominated responses.

### Study limitations

This study only had a small sample size as the focus was on the qualitative descriptions people used to provide reasons for their numerical ratings, rather than gathering a sample size sufficient for statistical testing. Authors were satisfied data saturation was reached as no new codes were being generated with the addition of the last few participants. Providing a “fatigue” scenario to participants may have helped set the situation a little better and made it easier to answer some of the questions. However, the lack of a scenario also aided the results being generalizable to a variety of situations as the responses highlighted how participants felt they would perceive the soundscape depending on a variety of situations, and thus the construct validity of the items across a variety of situations. These insights may have been lost if a fixed “fatigue scenario” had been provided to participants. The lack of personal pronouns in the Extent items for the PRSS interpretation questionnaire may have resulted in the strong emphasis on the physical environment without a consideration of the individual, compared to the other items. These were excluded due to the definitions focus on the environment rather than the individual's interpretation of the environment. Participants also seemed to interpret the items designed to measure Extent in this way too, however it is unknown if the inclusion of personal pronouns would have resulted in an individual perspective or if the concept of Extent does and should only relate to the “objective” physical characteristics of the environment. Ideally to assess the differences in the framing of the items holistically or specifically, the words “sounds” and “soundscapes” should have been a straight switch, but the order of the sentence was also reversed. Unfortunately, this resulted in some awkwardly read items which may have slightly affected comparisons between the holistic and specific framed items. The items were designed in this way to try and avoid a strong feeling of repetition for the participants. Half of the participants however still noticed the similarity of items “5, see no. 17” (*curiosity soundscape*) at least on one occasion. This suggests that regardless of the structure of the item, participants would have responded similarly anyway. Finally the study was conducted in one context, an indoor environment, thus differences may arise if conducted in a different environment such as outdoors. Such differences are expected to be minor, but further research could check this and determine if the interpreted themes remain consistent across each ART component in different environments.

## Conclusion

Through this qualitative study, which investigated the construction and interpretation of PRS, PRC and PRSS items, advancements in understanding the face and construct validity of the PRSS have occurred. In addition, theoretical and further methodological implications have arisen from the findings which are summarized below.

### PRSS face and construct validity

The PRSS was originally adapted from PRS and PRC scales which focus on all aspects of the environment rather than one sensory aspect, although the PRS and PRC have largely been used to rate elements in visual images. The PRSS has previously been tested in experimental and real world conditions where both visual and acoustical information was present (Payne, 2013; Evensen et al., 2016), as was the case in this study. Examination of participant responses suggest at times, participants were considering other information than just the sounds, although acoustic terms were used more frequently than visual terms. This highlights the difficulty in constructing a subjective measure for a singular sense when multiple sensory stimuli is available, particularly as evidence suggests one sense is strongly influenced by other sensory information (Bayne and Spence, 2015). This brings into question the value and validity of the PRSS when used in real world environments and potentially of other sound specific subjective measures. However, the PRSS still has value in laboratory settings where sensory stimuli can be systematically manipulated and the perceived restorativeness of different sounds and soundscapes can be monitored, including in interaction with other sensory stimuli. For example, the PRSS can differentiate between soundscapes within the same environment type, such as urban parks (Payne, 2010) and cafes as suggested in this study. This means that under controlled laboratory conditions where all other visual and contextual information remains the same, the PRSS could be a useful tool for helping designers to determine whether the addition of certain sounds, such as a fountain into a café, would be beneficial in creating an environment with greater perceived restorative qualities.

During the development of the PRSS interpretation questionnaire used in this study, differences in the vocabulary, grammar, and framing of PRSS, PRS, and PRC items were noted both within and between ART components. From this, two sets of items were developed, one set framed specifically (about sounds) and one set framed holistically (about soundscape). Results indicated that participant responses did not differ numerically or thematically between paired specific and holistic items.

Six themes were interpreted from participant justifications of numerical responses to PRSS items. Two related to the individual (personal attributes, personal outcomes), two related to the environment (physical environment attributes, soundscape design), and two were an interaction between individuals and the environment (behavior setting, normality/typicality). This mix of individual and environment themes is in line with ART which discusses both restorative environments and restorative experiences (Kaplan, 1995). Therefore the PRSS items appear to be engaging participants to think about all the necessary aspects for measuring perceived restorativeness, thus supporting construct validity. In addition, this study identified that respondents interpreted items measuring the different ART components in thematically different ways; ART component responses varied in the extent to which the individual, the environment, or the interaction between the individual and its environment was emphasized. This has implications for studies which choose to only use items that measure some of the ART components such as Fascination and Being-Away. In such studies, the environmental aspects and the interaction between the individual and environment may not be included as much in the perceived restorativeness rating, which may reduce the full understanding of a soundscape's restorative qualities. However, participants freely referred to the two main theoretical outcomes from restoration, recovery of attentional fatigue and reflection, which again supports the construct validity of the scale.

### Methodological implications for soundscape and restoration research

A number of wider methodological issues were raised from this study. First, many studies ask participants to rate a soundscape after a brief exposure time, lasting a few seconds or minutes. This study suggested that longer periods of exposure to a soundscape (around 40 min) can influence soundscape assessments, in particular for the ART components Fascination and Extent. Future studies should review suitable exposure times to ensure a fair assessment of all evaluative criteria. Secondly, for restoration research setting a fatigue scenario (and perhaps measuring baseline fatigue levels,) is important to avoid many of the “it depends” responses provided in this study. In this study the lack of a fatigue scenario was useful in highlighting the range of potential reasons people may use to respond to perceived restorativeness soundscape assessments, but fatigue scenarios are necessary for studies aiming to produce a restorativeness soundscape value. The type of fatigue scenario used should however be carefully considered, particularly if the environment can be used for a variety of activities. Indeed some of the responses in this study suggest different soundscapes may have different restorativeness values depending on the individual's type of attentional fatigue (such as work related or personal life issues). Thirdly, this study found assessing involuntary attention (Fascination) via self-reporting subjective statements problematic when the study task involves directing attention to the soundscape. Future studies may need to explore other means of assessing involuntary attention of soundscapes, such as through electroencephalogram scans (EEG), as an equivalent to the eye tracking studies starting to be used to assess Fascination in visual studies (Berto et al., 2008; Nordh et al., 2013).

### Theoretical implications for attention restoration

Broader ART implications also arose from the research. Minor adjustments or reemphasis to the definitions of Fascination and Compatibility are suggested to emphasize characteristics that are assumed from the interpretation of the current definitions or how they are currently measured. The positive quality of Fascination always needs noting, alongside an emphasis of the effortlessness of involuntary attention, as sounds can direct attention involuntarily, but sometimes in a draining and undesirable way (e.g., erratic banging from a neighboring construction site). Using explicit definitions will improve the accuracy of tools designed to measure the defined concept. A relationship between Compatibility and Preference often found in restorative environment research was also highlighted in these soundscape assessment responses, due to the words used in the Compatibility items. Examination of the statistical analysis of the relationship between Compatibility and preference scores in other studies is necessary to decide if there is a need to measure and assess *both* preference and compatibility in restorative soundscape research if they are highly related.

Compatibility was highlighted as an important ART component as more than any other component it led participants to specifically focus on the personal outcomes from experiencing the soundscape, including the two main outcomes said to derive from restorative experiences and environments—recovery and reflect. Extent was also identified to be particularly important for the perceived restorativeness of soundscapes and was particularly affected by the “objective” physical environment attributes rather than individual experiences. Extent is often neglected in restoration research, but this study suggests it may be particularly important for the restorativeness of soundscapes and key for considering the implications of soundscape design and the rest of the physical environment.

Restorative soundscapes are created through a combination of the physical environment and individuals' interpretation of that soundscape as restorative. This research suggests all four ART components are important to ensure soundscapes can be designed to create a potentially restorative environment and that people have a restorative experience, with each component contributing to understanding the environment, the individual, or a mixture of the two. To confirm these theoretical implications, further investigation into them would be necessary via examination of responses to the original PRS, rather than soundscape specific ones to ensure the implications related to broader theoretical aspects rather than sensory specific issues. Such work was conducted at the same time of this study but is not fully analyzed. Finally, as discussed by Aletta et al. (2016a) the relationship between restorativeness and other soundscape descriptors, such as pleasantness-eventfulness (Axelsson et al., 2010) and appropriateness (Brown et al., 2011) could be explored further to monitor any overlap.

## Author contributions

SP conceptualized the research question and study, and received the Post-Doctoral Fellowship funding support. SP conducted the data collection, coding and interpretation of themes, and is the primary author of the manuscript. CG assisted with the research development, coding and interpretation of items, and contributed to the paper manuscript.

### Conflict of interest statement

The authors declare that the research was conducted in the absence of any commercial or financial relationships that could be construed as a potential conflict of interest.

## Footnotes

^1^See Brown et al. (2011) for a review of previously used definitions and a description of ISO Working Group 54 - Assessment of soundscape quality.

^2^The term sonic environment was used rather than soundscape as initial comments from laypeople at the time was that the former was easier to comprehend.

## References

[B1] Aletta F. Kang J. Axelsson O. (2016a). Soundscape descriptors and a conceptual framework for developing predictive soundscape models. Landscape Urban Plan. 149, 65–74. 10.1016/j.landurbplan.2016.02.001

[B2] Aletta F. Lepore F. Kostara-Konstantinou E. Kang J. Astolfi A. (2016b). An experimental study on the influence of soundscapes on people's behaviour in an open public space. Appl. Sci. 6:276 10.3390/app6100276

[B3] Andringa T. C. Weber M. Payne S. R. Krijnders J. D. Dixon M. N. Lindon R.. (2013). Positioning soundscape reserach and management. J. Acoust. Soc. Am. 134, 2739–47. 10.1121/1.4819248 24116412

[B4] Axelsson O. Nilsson M. E. Berglund B. (2010). A principal components model of soundscape perception. J. Acoust. Soc. Am. 128, 2836–46. 10.1121/1.3493436 21110579

[B5] Barker R. G. (1965). Explorations in ecological psychology. Am. Psychol. 20, 1–14. 14251988 10.1037/h0021697

[B6] Bayne T. Spence C. (2015). Multisensory perception, in The Oxford Handbook of Philosophy of Perception, ed Matthen M. (Oxford: Oxford University Press), 603–620.

[B7] Bentler P. M. Jackson D. N. Messick S. (1971). Identification of content and style:a two-dimensionalinterpretation of acquiescene. Psychol. Bull. 76, 186–204. 10.1037/h0031474 4399323

[B8] Berto R. Massaccesi S. Pasini M. (2008). Do eye movements measured across high and low fascination photos differ? Addressing Kaplan's Fascination hypothesis. J. Environ. Psychol. 28, 185–191. 10.1016/j.jenvp.2007.11.004

[B9] Bild E. Steele D. Tarlao C. Guastavino C. Coler M. (2016). Sharing music in public spaces: social insights from the Musikiosk project (Montreal, CA), in INTER-NOISE and NOISE-CON Congress and Conference Proceedings (Hamburg), Vol. 253, 3657–3666.

[B10] Brown A. L. Kang J. A. Gjestland T. (2011). Towards standardization in soundscape preference assessment. Appl. Acoust. 72, 387–392. 10.1016/j.apacoust.2011.01.001

[B11] Carles J. L. Lopez-Barrio I. de Lucio J. V. (1999). Sound influence on landscape values. Landscape Urban Plan. 43, 191–200. 10.1016/S0169-2046(98)00112-1

[B12] Clark C. Sörqvist P. (2012). A 3-year update on the influence of noise on performance and behavior. Noise Health 14, 292–296. 10.4103/1463-1741.104896 23257580

[B13] Davies W. Adams M. D. Bruce N. Cain R. Jennings P. Poxon J. (2009). The positive soundscape project: a synthesis of results from many disciplines, in Inter-Noise (Ottawa, ON), 379.

[B14] Davies W. J. Adams M. D. Bruce N. S. Cain R. Carlyle A. Cusack P. (2013). Perception of soundscapes: an interdisciplinary approach. Appl. Acoust. 74, 224–231. 10.1016/j.apacoust.2012.05.010

[B15] Dubois D. (2000). Categories as acts of meaning: the case of categories in olfaction and audition. Cogn. Sci. Quart. 1, 33–66. Available online at: https://www.researchgate.net/publication/228707245_Categories_as_acts_of_meaning_The_case_of_categories_in_Olfaction_and_audition

[B16] Evensen K. H. Raanaas R. K. Fyhri A. (2016). Soundscape and perceived suitability for recreation in an urban designated quiet zone. Urban Forestry Urban Green. 20, 243–248. 10.1016/j.ufug.2016.09.003

[B17] Gaver W. W. (1993a). How do we hear in the world? Explorations in ecological acoustics. Ecol. Psychol. 5, 285–313.

[B18] Gaver W. W. (1993b). What in the world do we hear? An ecological approach to auditory event perception. Ecol. Psychol. 5, 1–29.

[B19] Ge J. Hokao K. (2005). Applying the methods of image evaluation and spatial analysis to study the sound environment of urban street areas. J. Environ. Psychol. 25, 455–466. 10.1016/j.jenvp.2005.10.003

[B20] Gidlöf-Gunnarsson A. Öhrström E. (2007). Noise and well-being in urban residential environments: the potential role of perceived availability to nearby green areas. Landscape Urban Plan. 83, 115–126. 10.1016/j.landurbplan.2007.03.003

[B21] Glaser B. G. (1965). The constant comparative method of qualitative analysis. Soc. Prob. 12, 436–445. 10.2307/798843

[B22] Guastavino C. (2006). The ideal urban soundscape: Investigating the sound quality of French cities. Acta Acust. United Acust. 92, 945–951. Available online at: http://www.ingentaconnect.com/contentone/dav/aaua/2006/00000092/00000006/art00013

[B23] Guastavino C. (2007). Categorization of environmental sounds. Can. J. Exp. Psychol. Revue Can. De Psychol. Exp. 61, 54–63. 10.1037/cjep2007006 17479742

[B24] Guastavino C. Katz B. F. G. Polack J. D. Levitin D. J. Dubois D. (2005). Ecological validity of soundscape reproduction. Acta Acust. United Acust. 91, 333–341. Available online at: http://www.ingentaconnect.com/contentone/dav/aaua/2005/00000091/00000002/art00015

[B25] Hartig T. Korpela K. M. Evans G. W. Gärling T. (1997). A measure of restorative quality in environments. Scand. Hous. Plan. Res. 14, 175–194. 10.1080/02815739708730435

[B26] Herzog T. R. Black A. M. Fountaine K. A. Knotts D. J. (1997). Reflection and attentional recovery as distinctive benefits of restorative environments. J. Environ. Psychol. 17, 165–170. 10.1006/jevp.1997.0051

[B27] Herzog T. R. Maguire C. P. Nebel M. B. (2003). Assessing the restorative components of environments. J. Environ. Psychol. 23, 159–170. 10.1016/S0272-4944(02)00113-5

[B28] ISO (2014). 12913-1:2014 Acoustics–Soundscape–Part 1: Definition and Conceptual Framework. ISO.

[B29] Kahn P. H. Jr Friedman B. B G. Hagman J. Severson R. L. Freier N. G. (2008). A plasma display window? *-* The shifting baseline problem in a technologically mediated natural world. J. Environ. Psychol. 28, 192–199. 10.1016/j.jenvp.2007.10.008

[B30] Kang J. Aletta F. Gjestland T. T. Brown L. A. Botteldooren D. Schulte-Fortkamp B. (2016). Ten questions on the soundscapes of the built environment. Build. Environ. 108, 284–294. 10.1016/j.buildenv.2016.08.011

[B31] Kaplan R. Kaplan S. (1989). The Experience of Nature: A Psychological Perspective. (Cambridge: Cambridge University Press).

[B32] Kaplan S. (1995). The restorative benefits of nature: toward an integrative framework. J. Environ. Psychol. 15, 169–182. 10.1016/0272-4944(95)90001-2

[B33] Korpela K. M. Hartig T. (1996). Restorative qualities of favourite places. J. Environ. Psychol. 16, 221–233. 10.1006/jevp.1996.0018

[B34] Laumann K. Gärling T. Stormark K. M. (2001). Rating scale measures of restorative components of environments. J. Environ. Psychol. 21, 31–44. 10.1006/jevp.2000.0179

[B35] Lindal P. J. Hartig T. (2013). Architectural variation, building height, and the restorative quality of urban residential streetscapes. J. Environ. Psychol. 33, 26–36. 10.1016/j.jenvp.2012.09.003

[B36] Moore B. C. J. (2003). An Introduction to the Psychology of Hearing. San Diego, CA: Academic Press.

[B37] Nordh H. Hagerhall C. M. Holmqvist K. (2013). Nordh_tracking restorative components patterns in eye movements as a consequence of a restorative rating task. Landscape Res. 38, 101–116. 10.1080/01426397.2012.691468

[B38] Nordh H. Hartig T. Hagerhall C. M. Fry G. (2009). Components of small urban parks that predict the possibility for restoration. Urban Forest. Urban Green. 8, 225–235. 10.1016/j.ufug.2009.06.003

[B39] Pals R. Steg L. Siero F. van der Zee K. I. (2009). Development of the PRCQ: a measure of perceived restorative characteristics of zoo attractions. J. Environ. Psychol. 29, 441–449. 10.1016/j.jenvp.2009.08.005

[B40] Payne S. R. (2008). Are perceived soundscapes within urban parks restorative?, in Proceedings of Acoustics '08 (Paris).

[B41] Payne S. R. (2010). Urban park soundscapes and their perceived restorativeness, in Proceedings of the Institute of Acoustics and Belgium Acoustical Society (Ghent), Vol. 32, 264–271.

[B42] Payne S. R. (2013). The production of a Perceived Restorativeness Soundscape Scale. Applied Acoustics 74, 255–263. 10.1016/j.apacoust.2011.11.005

[B43] Payne S. R. Guastavino C. (2013). Measuring the perceived restorativeness of soundscapes: is it about the sounds, the person, or the environment?, in INTER-NOISE and NOISE-CON Congress and Conference Proceedings (Innsbruck), Vol. 247, 2223–2229.

[B44] Purcell T. Peron E. Berto R. (2001). Why do preferences differ between scene types? Environ. Behav. 33, 93–106. 10.1177/00139160121972882

[B45] Raimbault M. Lavandier C. Berengier M. (2003). Ambient sound assessment of urban environments: field studies in two French cities. Appl. Acoust. 64, 1241–1256. 10.1016/S0003-682X(03)00061-6

[B46] Schoggen P. (1989). Behaviour Settings: A Review and Extension of Roger G Barker's Ecological Psychology. Stanford, CA: Stanford University Press.

[B47] Scott M. J. Canter D. V. (1997). Picture of place? A multiple sorting study of landscape. J. Environ. Psychol. 17, 263–281.

[B48] Staats H. Kieviet A. Hartig T. (2003). Where to recover from attentional fatigue: An expectancy-value analysis of environmental preference. J. Environ. Psychol. 23, 147–157. 10.1016/S0272-4944(02)00112-3

[B49] Stansfeld S. A. Berglund B. Clark C. López-Barrio I. Fischer P. Öhrström E.. (2005). Aircraft and road traffic noise and children's cognition and health: a cross national study. Lancet 365, 1942–1949. 10.1016/S0140-6736(05)66660-3 15936421

[B50] Steffens J. Steele D. Guastavino C. (2017). Situational and person-related factors influencing momentary and retrospective soundscape evaluations in day-to-day life. J. Acoust. Soc. Am. 141, 1414–1425. 10.1121/1.4976627 28372095

[B51] Tennent R. Hiller L. Fishwick R. Platt S. Joseph S. Weich S. (2007). The Warwick-Edinburgh Mental Well-Being Scale (WEMWBS): development and UK validation. Health Qual. Life Outcomes 5:63 10.1186/1477-7525-5-63 18042300 PMC2222612

[B52] Ulrich R. S. Simons R. F. Losito B. D. Fiorito E. Miles M. A. Zelson M. (1991). Stress recovery during exposure to natural and urban environments. J. Environ. Psychol. 11, 201–230. 10.1016/S0272-4944(05)80184-7

[B53] Van Kamp I. Klæboe R. Brown A. L. Lercher P. (2015). Soundscapes, human restoration and quality of life, in Soundscape and the Built Environment (Boca Raton, FL: CRC Press), 43–68.

[B54] World Health Organisation (2011). Burden of Environmental Noise: Quantification of Healthy Life Years Lost in Europe. Copenhagen: World Health Organisation.

